# Community composition and trophic mode diversity of fungi associated with fruiting body of medicinal *Sanghuangporus vaninii*

**DOI:** 10.1186/s12866-022-02663-2

**Published:** 2022-10-19

**Authors:** Yanjun Ma, Weiqian Gao, Fan Zhang, Xuetai Zhu, Weibao Kong, Shiquan Niu, Kun Gao, Hongqin Yang

**Affiliations:** College of Life Sciences, Northwest Normal University, 730070 Lanzhou, Gansu, China

**Keywords:** *Sanghuangporus* fruiting body, Fungal diversity, Functional group, High throughput sequencing

## Abstract

**Background:**

The microbial symbionts of macrofungal fruiting body have been shown to play momentous roles in host growth, development, and secondary metabolism. Nevertheless, there is no report on the fungal diversity of *Sanghuangporus*, a medicinal and edible homologous macrofungus as “forest gold”, which has good effects on antioxidation, boosting immunity and curing stomachache. Here, the diversity and functional group of fungi associated with the fruiting body of the most widely applied *S. vaninii* were characterized by high-throughput sequencing and FUNGuild tool for the first time.

**Results:**

Total 11 phyla, 34 classes, 84 orders, 186 families, and 328 genera were identified in the fruiting body, and our results revealed that the fungal community was dominated by the host fungal taxonomy with absolute superiority (more than 70%), namely, Basidiomycota, Agaricomycetes, Hymenochaetales, Hymenochaetaceae, and genus of *Phellinus* corrected to *Sanghuangporus*. Simultaneously, the reads allocated into non-host fungal operational taxonomic units were largely dominated by Ascomycota, Sordariomycetes, Sordariales, Mortierellaceae, and *Mortierella*. Furthermore, the endophytic fungi were assigned into three trophic modes of “saprotroph” (53.2%), “symbiotroph” (32.2%), and “pathotroph” (14.1%), in which the category of “plant pathogen” was highest enriched with relative abundance of 91.8%, indicating that the endophytic fungi may have the potential to adjust the growth and metabolism of host *S. vaninii*.

**Conclusion:**

Altogether, this report firstly provided new findings that can be inspiring for further in-depth studies to exploit bioactive microbial resources for increased production of *Sanghuangporus* via coculture, as well as to explore the relationship between macrofungi and their associated endophytes.

**Supplementary Information:**

The online version contains supplementary material available at 10.1186/s12866-022-02663-2.

## Introduction

Mushroom-forming macrofungi are momentous natural resources of food and medicine in the market owing to the various and affluent metabolites [1–3], among which the famous “Sanghuang” has been used in Traditional Chinese Medicine (TCM) for the treatment of stomachache, hepatopathy and gynecological disorders for the past two centuries [4]. Unfortunately, “Sanghuang” used to be incorrectly called *Phellinus linteus*, *Inonotus sanghuang*, *P. baumii*, and *P. igniarius* for many years. “Sanghuang” and its similar species were classified into Basidiomycota, Agaricomycetes, Hymenochaetales, Hymenochaetaceae, and a new genus of “*Sanghuangporus* Sheng H. Wu, L.W. Zhou & Y.C. Dai” by multigene fragment-based phylogenetic analysis until 2015 [5]. Simultaneously, 14 species of *Sanghuangporus* fungi exist throughout the world, in which *S. vaninii* can be widely artificially cultivated and be also far more popular with customers in China [6, 7]. Given the insufficiency of excellent strains, long growth cycle, dim formation mechanism, and over exploitation of fruiting body [8], the production of *Sanghuangporus* consequently still could not meet the requirements of the market.

Owing to the abundant bioactive metabolites including phenolic, flavonoid, polysaccharide, etc. and their conspicuous pharmacological activities such as antitumor, antioxidant, anti-inflammatory, and antimicrobe abilities [9–12], the fungus *Sanghuangporus* of “forest gold” has attracted the extensive attention of researchers. In addition, it was found that the contents of active compounds and their biological activities in artificial cultivated fruiting bodies of *Sanghuangporus* were higher and stronger than those in wild ones [13, 14]. Therefore, several strategies were applied into the mycelium cultures or fruiting body cultivation to improve the efficiency and quality of *Sanghuangporus* production, including mutation breeding [15], the optimization of medium composition [16], the improvement of cultural condition [17], and the addition of exogenous fungal elicitor [18]. It was worth mentioning that the growth and development of both wild and cultivated macrofungal fruiting bodies were in association with various endophytic microorganisms, in which the community structures and roles of bacteria inhabiting in fruiting bodies have been widely explored and reported. As early as 1991, it was observed that *Pseudomonas putida* isolated from the fruiting body of *Agaricus bisporus* could stimulate the extension of host mycelia but restrain the hyphal branching frequency [19]. In recent years, Oh et al. (2018) reported the isolates *Dietzia*, *Ewingella*, *Pseudomonas*, *Paenibacillus* and *Rodococcus* were able to promote the growth of host pine mushroom (*Tricholoma matsutake*), whereas the remaining bacteria including *Mycetocola* and *Stenotrophomonas* had the negative impacts on host growth irrelevant to their various enzyme activities such as chitinase, cellulase, and protease [20]. Our previous research revealed that *Bacillus* and *Pseudomonas* were the predominant taxa of host *Shiraia* fruiting body, in which the *Pseudomonas* isolates could stimulate the production of photosensitive drug (hypocrellin A) extracted from *Shiraia* [21]. In addition, the morphogenesis of fruiting bodies of mushroom was actually accompanied by the existence of a variety of neglected fungicolous fungi, which were reported for the first time in Japan [22]. Afterward, several truffle-inhabiting fungi including filamentous fungi and yeasts isolated from the fruiting bodies such as *Tuber melanosporum*, *T. magnatum*, etc., were able to secret volatile organic compounds or to regulate mycorrhizal synthesis [23–25], but far less is known about the diversity and richness of endophytic fungi associated with mushroom. Only in recent years, studies have shown that the phyla of Basidiomycota, Ascomycota, and Mucoromycota were detected besides host fungus *T. aestivum* by using the Illumina MiSeq method [25]. Maurice et al. (2021) reported that the non-host ITS2 reads of endophytic fungi inhabiting in the 176 fungal sporocarps collected within a same forest were largely dominated by Ascomycota by high-throughput sequencing [26]. Nevertheless, the presence and potential role of fungi associated with *Sanghuangporus* fruiting body also remain unknown.

Against this background, we herewith wish to investigate the fungal community structure of cultivated fruiting body of the most popular *S. vaninii* by means of high-throughput sequencing, which has become a powerful means to provide more rapid and distinguishable analysis on the microbial communities including predominant groups, rare clusters, and even some unknown species compared with the culture-dependent method [21, 24, 26]. For instance, our previous experimental data displayed *Bacillus* and *Pseudomonas* with higher frequency of occurrence based on both culture-dependent and next-generation sequencing approaches, whereas other members of *Paenibacillus* and *Sphingomonas* were common in Illumina sequences but scarce in isolation cultures [21]. To our best knowledge, this is the first attempt of investigation on the diversity of fungi associated with *Sanghuangporus* fruiting body, contributing to a better understanding of the diversity and functional attribute of fungicolous fungi.

## Materials and methods

### Sampling and DNA extraction

**Fig. 1:**
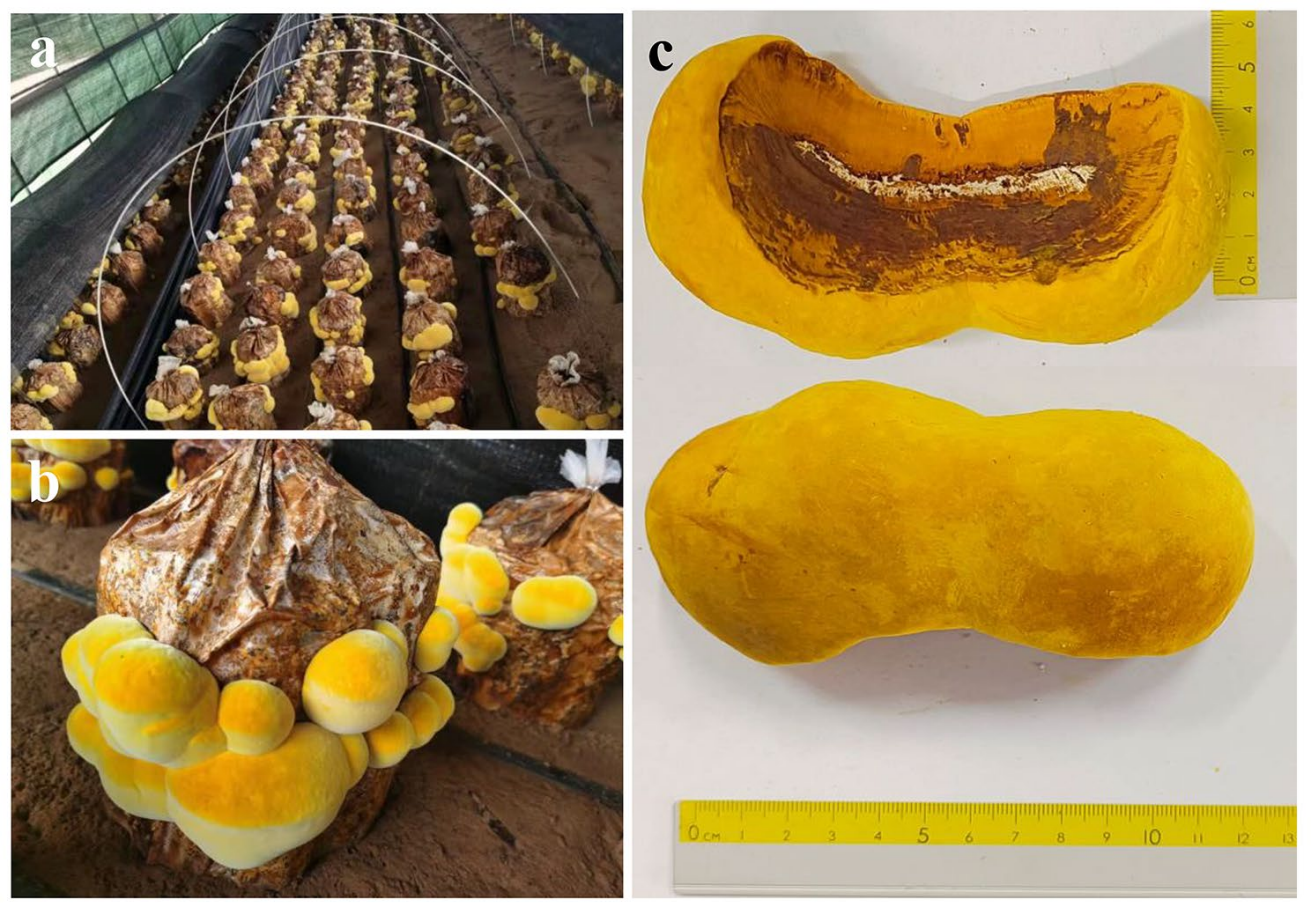
The morphology of *S. vaninii* fruiting bodies. **a** Industrialized cultivation of fruiting bodies in the Mahe Industrial Park of Edible Fungi of Yulin. **b** The fruiting body on the artificial substrates. **c** Collected fruiting bodies

The fruiting bodies of *S. vaninii* were collected from the bag-cultivated surface at their mature stage from September to October 2021, in Mahe Industrial Park of Edible Fungi of Yulin (Fig. 1), China and picked from three plots (approximately 50 bags/plot). Each set of samples (SH1, SH2, and SH3) contained 10 fruiting bodies. All macrofungal samples were promptly deposited in microbe-free sacks and transferred to the laboratory for subsequent analysis.

Fresh fruiting bodies of *S. vaninii* were sterilized according to our previous report with slight modification [21]. In short, the fruiting bodies were sterilized by immersion in 0.1% HgCl_2_ (w/v) for 1 min and 75% ethanol for 30 s, and then washed with aseptic water for 5 times and dried with sterile tissue. In order to test the efficiency of fruiting body surface sterilization, an essential control experiment was set up by coating sterile distilled H_2_O (200 uL) derived from the last step of surface sterilization on fungal isolation media in common use, such as potato dextrose agar and Martin medium. No microorganism growth was discovered on the medium plates after 8 days of incubation at 28-30^o^C, demonstrating that above sterilization means was successful in killing or at least inhibiting the growth of the epiphytic or environmental microbes [27–29]. After that, 90 small pieces (approximately 3 × 1 × 1 cm each) as a group, were evenly cut from the ten fruiting bodies of each sample (SH1, SH2, and SH3) by sterile scissors. The cutting pieces of above three groups were separately ground into powder with liquid nitrogen by sterilized 12-cm mortar and pestle, and then placed on ice for standby. Then, the total genomic DNA of the associated fungi were extracted from above powders (200 mg for each group) of fruiting body by using TIANamp Plant Genomic DNA Kit (Tiangen-Biotech, Beijing, China) according to the manufacturer’s protocol. The high qualities including the integrity of electrophoresis bands, and the purity (OD_260_/OD_280_ = 1.8-2.0) and concentration (20-30 mg/100 mg sample) of DNA specimens were measured by using Agilent 2100 Bioanalyzer (Agilent Technologies, Santa Clara, CA, United States) and then sealed at -20^o^C.

### Polymerase chain reaction (PCR) amplification and deep sequencing

The fungal-specific primers ITS1F (5’-CTTGGTCATTTAGAGGAAGTAA-3’) and ITS2R (5’-GCTGCGTTCTTCATCGATGC-3’) were used to amplify the ITS1 region of ITS rDNA gene [30]. The PCR reactions were carried out in a final volume of 50 µL, comprising 100 ng of template DNA, 25 µL of Phusion Hot start flex 2× Master Mix, and 2.5 µL of 10 µmol L^-1^ each forward and reverse primers (ITS1F/ITS2R), made up to the final volume with double distilled water (dd H_2_O). The same volume of ddH_2_O instead of template DNA was added to above PCR system as a negative control group. The PCR reaction of ITS1 rDNA was implemented under the following procedures reported by Thijs et al. (2017) [31] with a minor modification: 3 min at 94^o^C, followed by 25 cycles of 60 s at 95^o^C, 60 s at 50^o^C, and 60 s at 72^o^C, and then a final 7 min extension step at 72^o^C was executed using T-100 thermal cycler (Bio-Rad, Hercules, CA, United States).

Above PCR products were subsequently corroborated by 2% (w/v) agarose gel. The DNA bands with the correct size were excised and purified using AMPure XT beads (Beckman Coulter Genomics, Danvers, MA, United States), whose correct size was about 500 base pairs (bp) equaled to the PCR products (approximately 400 bp) of ITS plus the sequencing connector (approximately 100 bp) of gel recovered products. After purification, the PCR products of ITS1 rDNA regions were quantified using Qubit system (Invitrogen, USA). The libraries were then assessed by Agilent 2100 Bioanalyzer and sequenced by high-throughput Illumina NovaSeq PE250 platform (Illumina, San Diego, CA, United States) [32]. At present, the Illumina sequencing raw data of endophytic fungi of *S. vaninii* fruiting body were submitted into the National Center for Biotechnology Information database (NCBI, https://www.ncbi.nlm.nih.gov/genbank/) with the SRA accession number of PRJNA820174.

### Bioinformatic processing and analysis of the sequencing data

The resulting 239,569 raw sequences were merged using the method reported by Zhang et al. (2014) [33], filtered of quality using Trimmomatic (version 0.33) [34], removed of forward and reverse primers using Cutadapt (version 1.9.1) [35], spliced of paired-end (PE) reads with FLASH (version 1.2.11) [36], and then removed of chimeras using UCHIME (version 8.1) [37] to obtain high-quality reads. Then, the clean reads were demodulated by the aid of DADA2 to obtain signature sequences [38]. The high-quality sequences were clustered into operational taxonomic units (OTUs) defined at 97% similarity using UPARSE pipeline [39]. Compared with SILVA database (https://www.arb-silva.de/), all OTUs were sorted out by using the plug feature-classifier of QIIME2 [40], which was also applied to analyze the rarefaction curve and alpha diversity of the samples, such as ACE, Chao1, and Simpson indices, etc. In addition, the Fungi Functional Guild (FUNGuild, http://www.stbates.org/guilds/app.php) was applied to identify the functional groups of endophytic fungal community [41]. In brief, all fungal OTUs at genus level (865, Supplementary Table S1) were submitted into FUNGuild and then widely divided into three trophic modes based on the Confidence of highly probable and probable assigned according to the primary research literatures or authoritative websites, which reflected the likelihood that a taxon belongs to a given guild [41].

### Statistical analysis

The calculation and comparative analysis of alpha diversity (OTU richness and index including ACE, Chao1 and Simpson) between three groups of samples were carried out by QIME2 (version 2020.06), and then the drawing was accomplished using R package (version 3.5.2). The calculation formula of Coverage was as follows: *C* = *1* - (*n*_*1*_/*N*), where *C* represents the Good’s coverage, *n*_*1*_ represents the number of OTUs with only one sequence, and *N* represents the total number of sequences in the sample draw.

## Results

### Metadata and sequencing statistics of ITS rDNA of *S. vaninii* fruiting body

In the industrialized artificial cultivation of *S. vaninii* (Fig. 1a), the fruiting bodies in the mature stage presented a bright yellow surrounding the surface of cultivated bags containing mulberry sawdust as raw material matrix (Fig. 1b). The fresh fruiting body was hard fleshy and compact with convex in the middle region, and the lignification degree of the interior was deeper than that of outside (Fig. 1c). Moreover, the average length of collected fruiting body was about 12-15 cm, the width was 4-6 cm, and the height was 2-4 cm with a dumbbell-like shape (Fig. 1c).

**Fig. 2:**
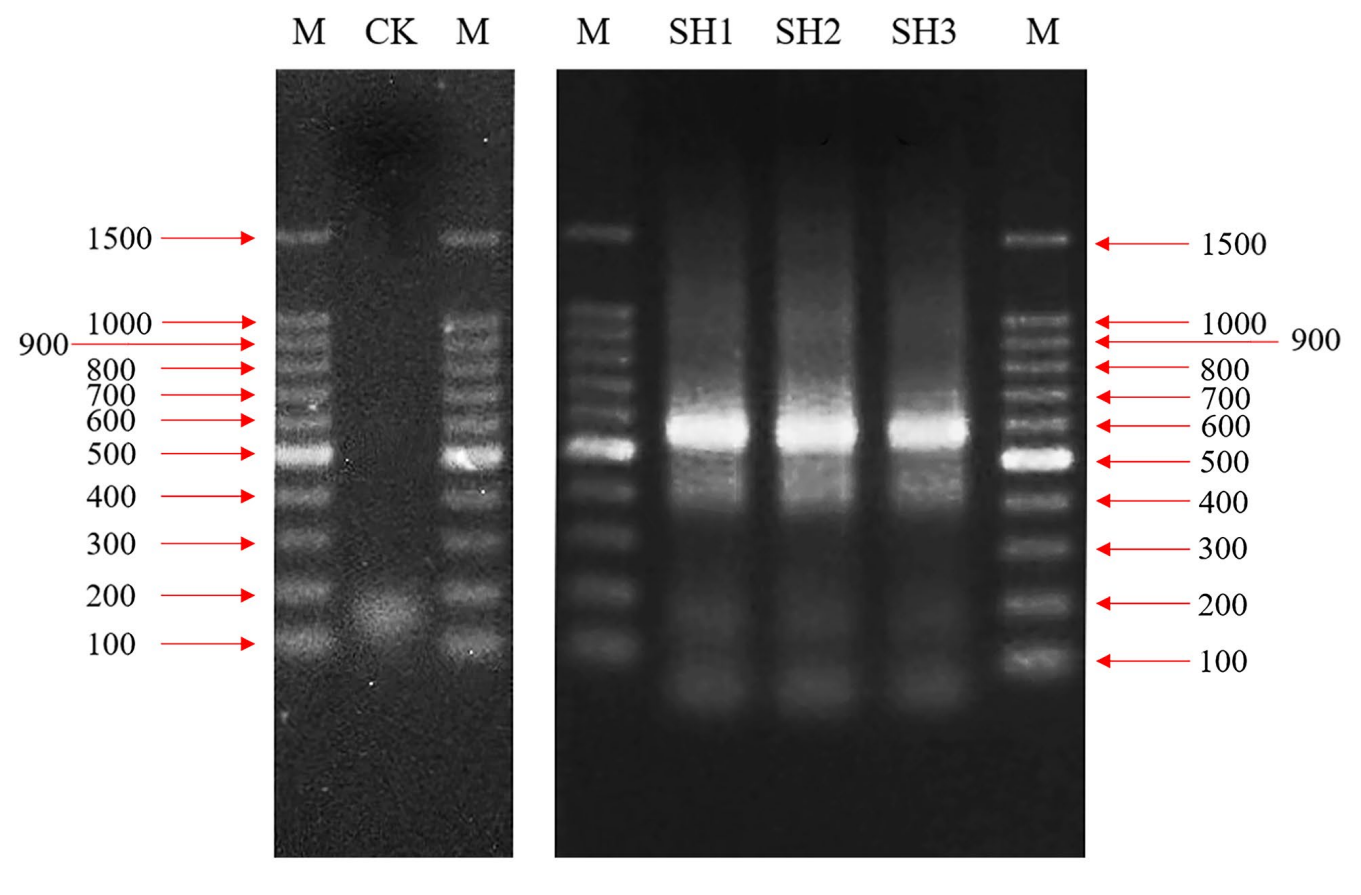
The electrophoretogram of PCR products of ITS rDNA extracted from the fruiting bodies of *S. vaninii*. M indicates the marker (DNA Ladder). CK indicates the H_2_O-treated group as negative control. SH1-3 indicate the experimental samples

**Fig. 3:**
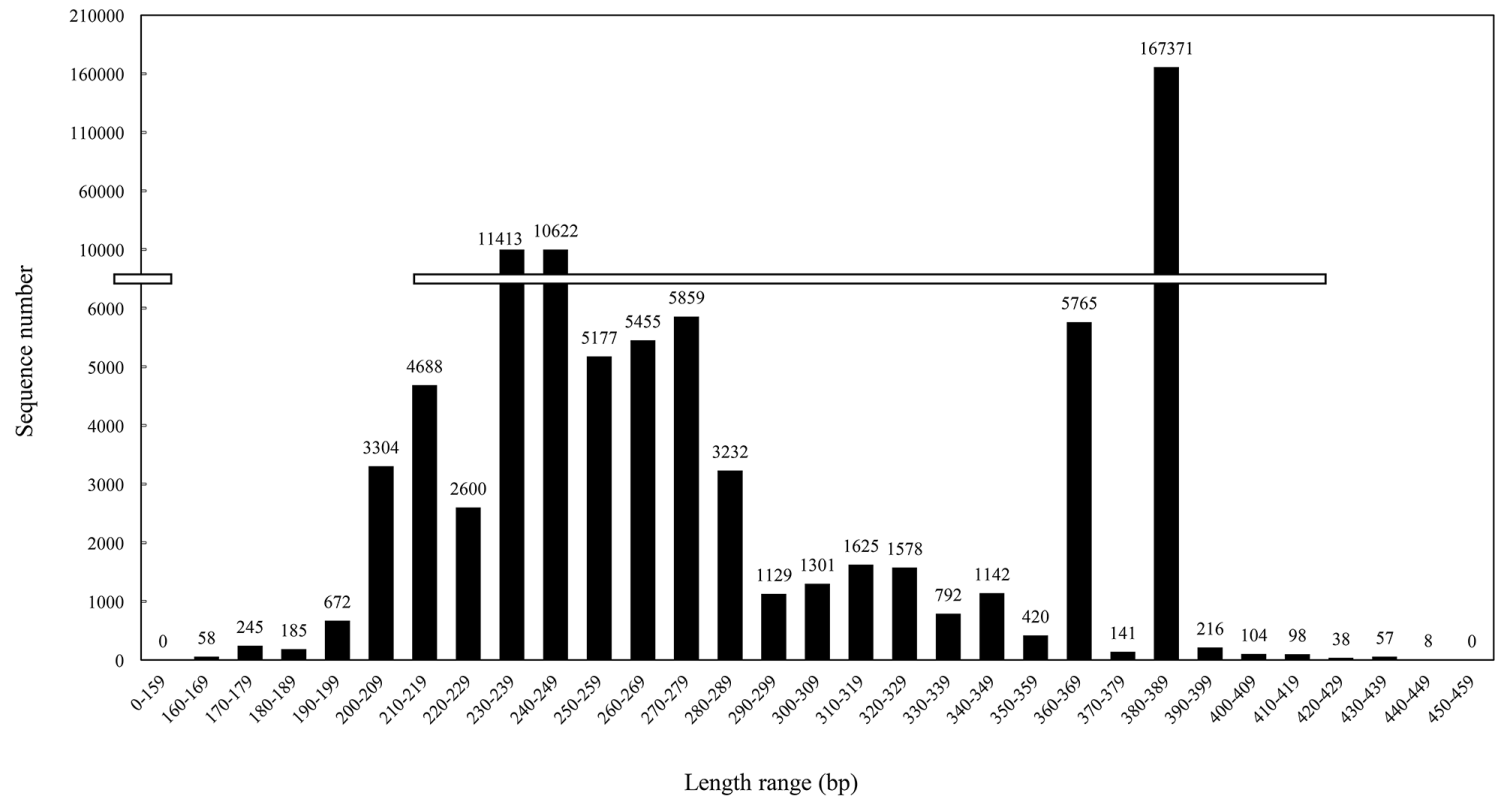
The length distribution of ITS rDNA sequences of fungi associated with *S. vaninii* fruiting body

First and foremost, the banding-free lane (CK in Fig. 2) of negative control demonstrated that the ITS sequences amplified by PCR stemmed from the *Sanghuangporus* samples rather than the pollution of laboratory environment or reagents. After the electrophoretic gel running (Fig. 2), the bioinformatic pipelines detected 238,410 clean reads were assigned to 865 OTUs (Table 1). And, the clean reads possessed an average length (AvgLen) of 346 bp, GC content of 48.0% and Q20 value of 99.4% (Table 1), indicating the high quality and accuracy of sequencing data in this research. It was noted that a large number of ITS rDNA reads were concentrated in the range of 380-389 bp (71.1%), even though some shorter reads (190-369 bp) were widely distributed (Fig. 3).

**Table 1:** Sequence and diversity statistics of fungal ITS1 rDNA genes obtained from *S. vaninii* fruiting body

Sample name	Clean reads ^a^	OTU ^b^	AvgLen (bp) ^c^	GC (%) ^d^	Q20 (%) ^e^
SH1	79,675	680	351	48.1	99.3
SH2	79,467	715	339	47.8	99.4
SH3	79,268	663	348	48.0	99.4
Total/Avg	238,410	865	346	48.0	99.4

^a^ The number of total reads that passed quality control

^b^ Total operational taxonomic unit

^c^ The average length of sequences

^d^ The percentage of G and C in all bases

^e^ The percentage of total number of bases where the Phred score is greater than 20 which indicates base call accuracy

### Community structures of fungi associated with *S. vaninii* fruiting body

**Fig. 4:**
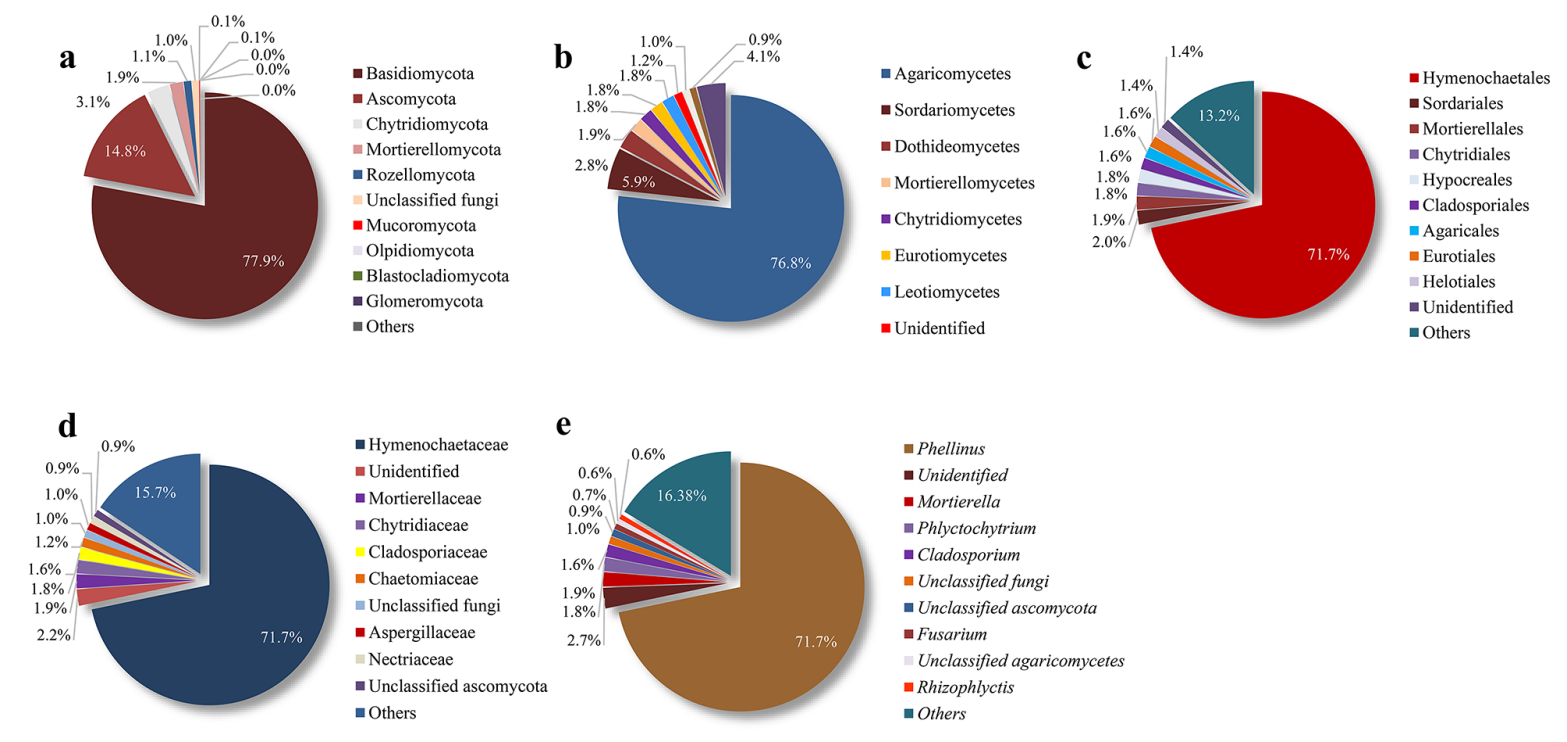
Relative abundance of fungal OTUs at **a** phylum, **b** class, **c** order, **d** family and **e** genus levels in *S. vaninii* fruiting body, respectively

As shown in Table 2 and Supplementary Table S1, the 865 fungal OTUs belonged to 11 phyla, 34 classes, 84 orders, 186 families, and 328 genera. Our experimental specimen of *S. vaninii* itself belonged to the phylum of Basidiomycota (78.0%), class of Agaricomycetes (76.8%), order of Hymenochaetales (71.8%), and family of Hymenochaetaceae (71.7%), were indeed dominant with absolute superiority of relative abundances in community structure (Fig. 4a-d).

**Table 2:** The OTU taxonomy of fungal community composition of *S. vaninii* fruiting body

Sample name	Kindom	Phylum	Class	Order	Family	Genus
SH1	1	11	32	77	170	283
SH2	1	10	30	80	168	291
SH3	1	11	33	77	168	277
Total	1	11	34	84	186	328

In addition, there were two main phyla in the OTUs including Ascomycota (14.8%), Chytridiomycota (3.1%), and some other groups with a small percentage (< 3.0%, Fig. 4a). There were two main classes including Sordariomycetes (5.9%), Dothideomycetes (2.8%), and some small proportion (< 2.0%) and unidentified ones (Fig. 4b). There were eight main orders from Helotiales with a relative abundance of 1.4% to Sordariales with 2.0% shown in Fig. 4c. There were four main families including Mortierellaceae (1.9%), Chytridiaceae (1.8%), Cladosporiaceae (1.6%), 1.2% Chaetomiaceae, and other small-percentage ones (< 1.0%, Fig. 4d). Among these fungi associated with the fruiting bodies, the genus of *Phellinus* (corrected to *Sanghuangporus* in 2015 by Zhou et al. [5]) was the most predominant fungi (71.7%), followed by *Mortierella* (1.9%), *Phlyctochytrium* (1.8%), *Cladosporium* (1.6%), *Fusarium* (0.7%), and *Rhizophlyctis* (0.6%) (Fig. 4e).

### Richness and diversity indices of associated fungi

**Fig. 5:**
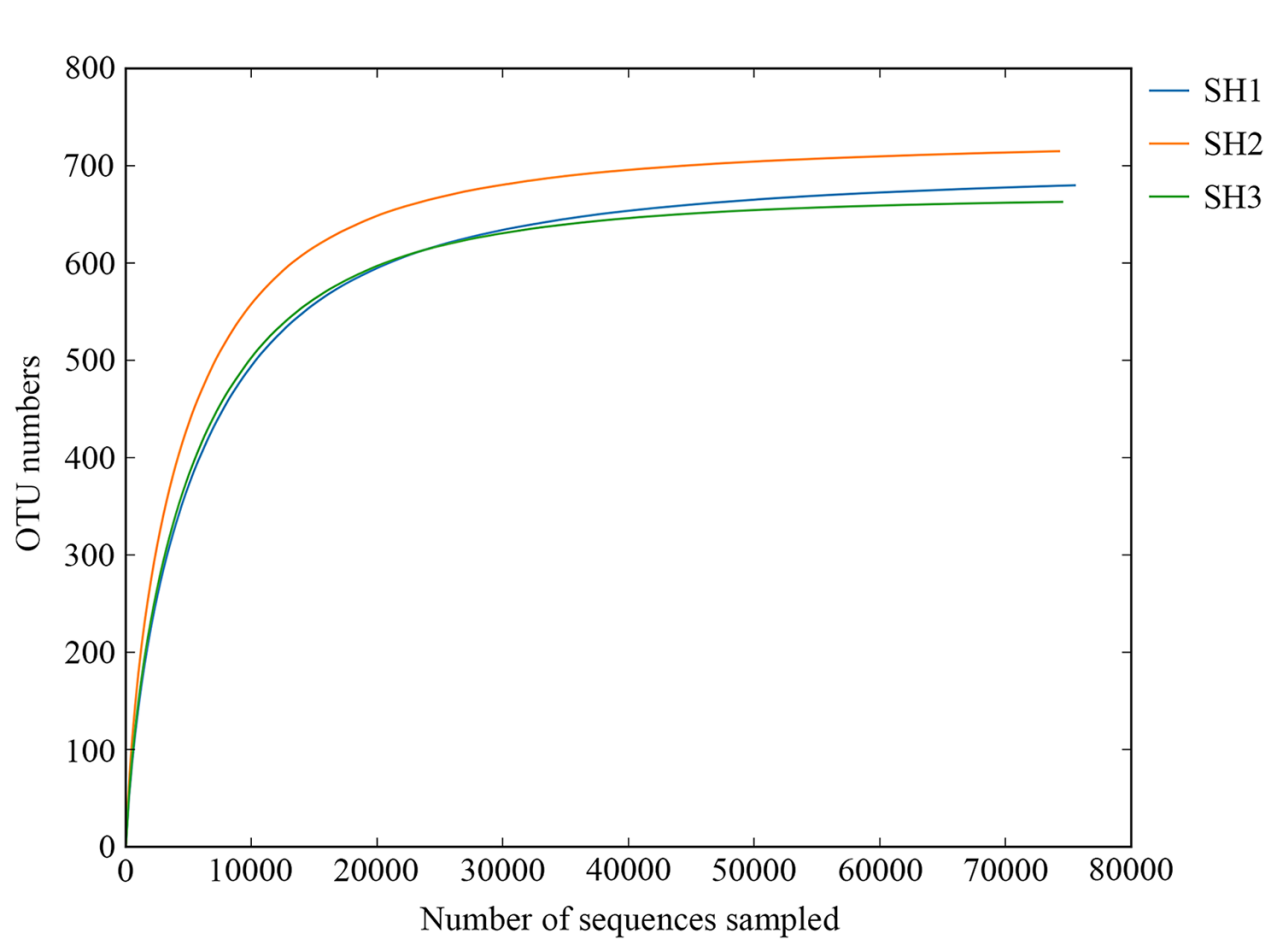
Rarefaction curve of detected OTUs of ITS rDNA at 3% dissimilarity level in *S. vaninii* fruiting body

As shown in Fig. 5, the rarefaction curve of all samples (SH1, SH2, and SH3) inclined to attain saturation, indicating that the data volume of sequences was sufficient in this analytical experiment. The values of richness indices including ACE and Chao1 representing species abundance, Simpson and Shannon representing species diversity, and Good’s Coverage representing the proportion of the detected species covering the actual ones are all presented in Table 3, demonstrating that the sequencing results could reflect the true diversity of fungi derived from all samples of *S. vaninii*.

**Table 3:** Richness estimators and diversity indices at a 97% identity threshold

Sample name	ACE	Chao 1	Simpson	Shannon	Good’s Coverage
SH1	687.8039	695.037	0.7057	3.4793	0.9996
SH2	720.2198	723.077	0.7759	4.2462	0.9997
SH3	665.9282	666.500	0.7228	3.6365	0.9998

### Fungi functional groups

**Fig. 6:**
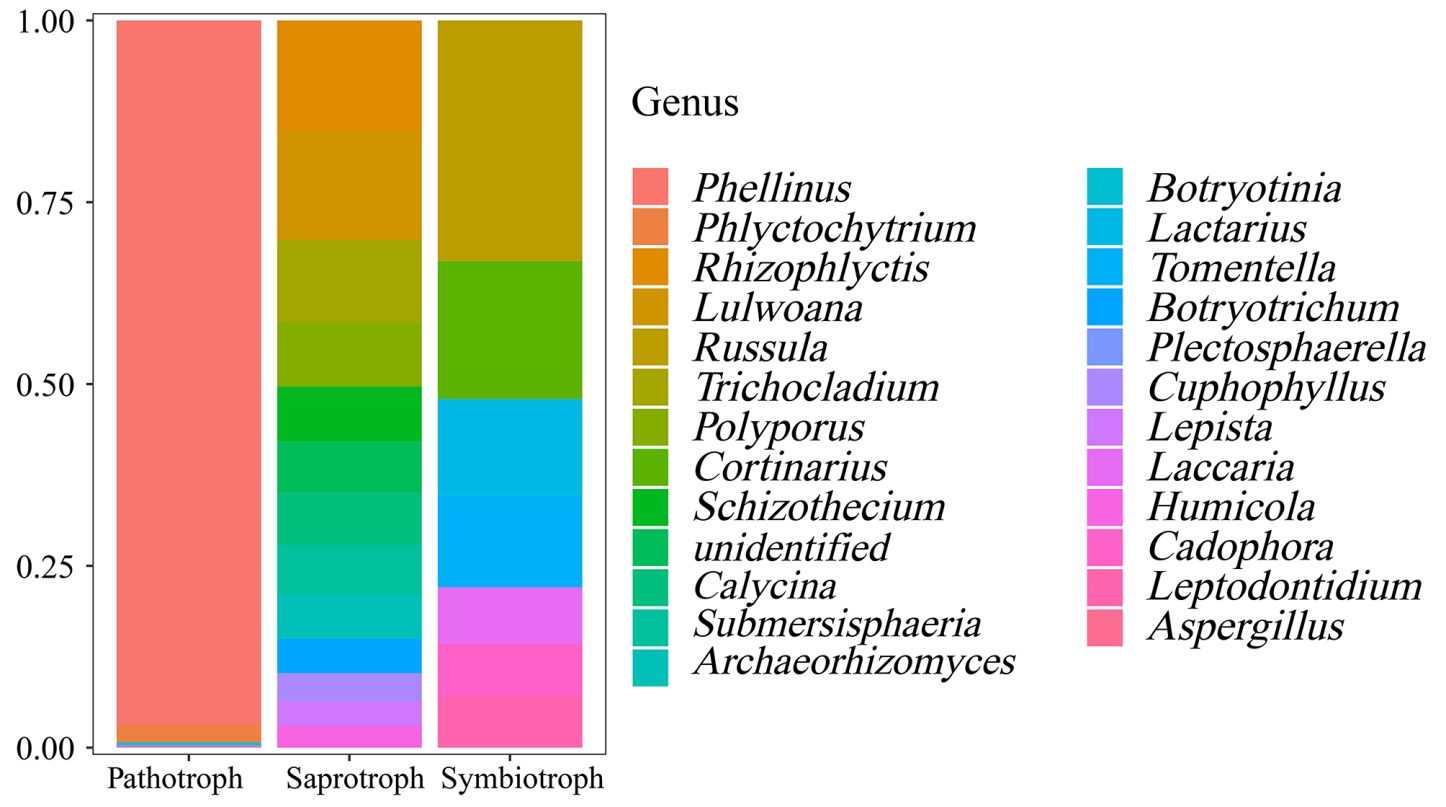
Trophic modes annotation of associated fungi of *S. vaninii* fruiting body at genus level

**Fig. 7:**
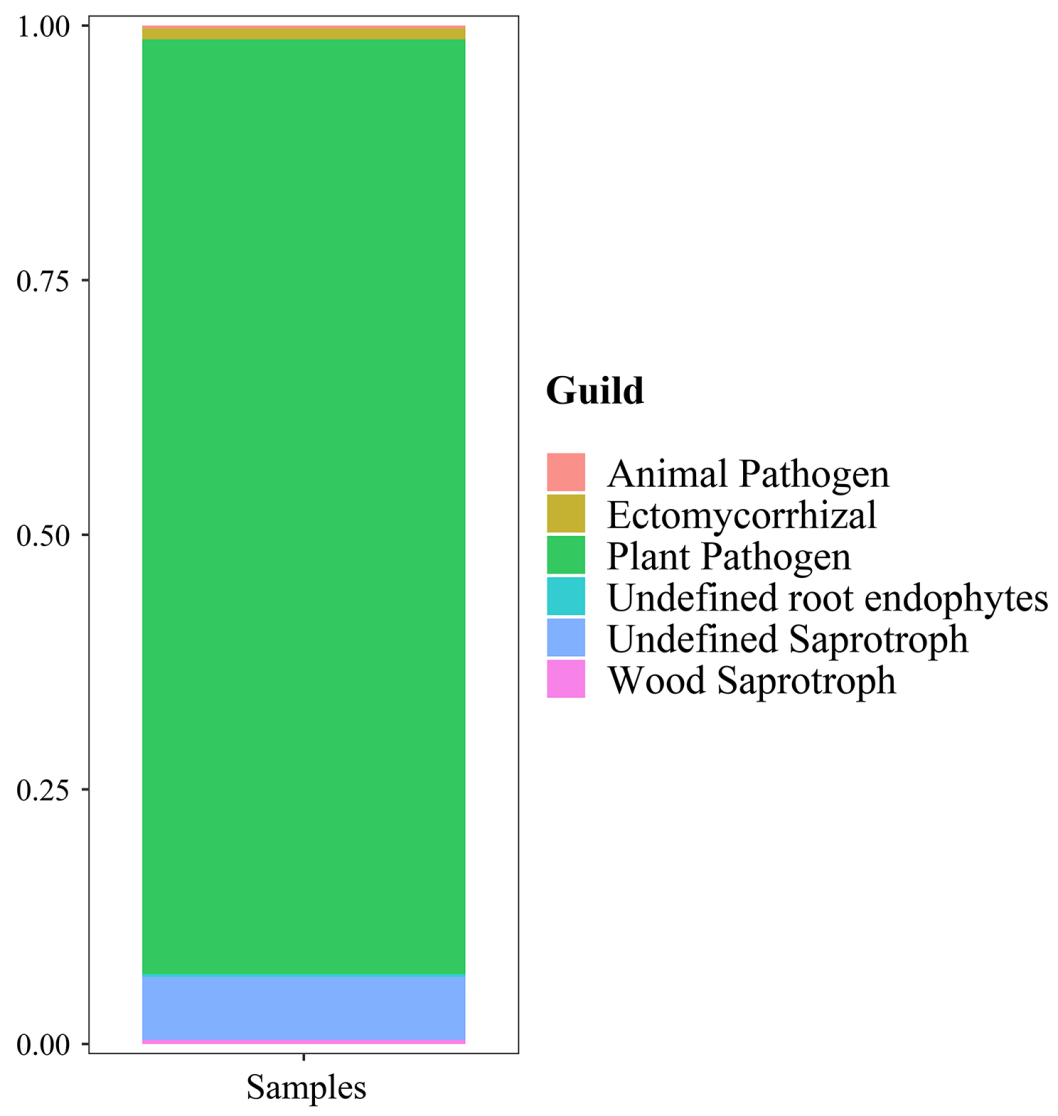
Trophic modes and their guilds groups of fungi associated with *S. vaninii* fruiting body at genus level

A total of 451 OTUs (52.1%) can be divided into trophic modes with “pathotroph”, “saprotroph” and “symbiotroph”, in which “saprotroph” was highest enriched with relative abundance of 53.2% (Supplementary Table S2). On the basis of Confidence of highly probable, the 25 genera of endophytic fungi with the highest abundance (Supplementary Table S3), namely Top25, were then selected and visualized with trophic mode as horizontal ordinate and relative abundance as longitudinal coordinate (Fig. 6). And specifically, the most dominant *Phellinus* (96.9%), followed by *Phlyctochytrium*, and other non-dominant fungi including *Botryotinia*, *Plectosphaerella*, and *Aspergillus* were only related to “pathotroph”. The dominated *Rhizophlyctis* and other non-dominant fungi including *Lulwoana*, *Trichocladium*, *Polyporus*, *Schizothecium*, *Calycina*, *Submersisphaeria*, *Archaeorhizomyces*, *Botryotrichum*, *Cuphophyllus*, *Lepista*, and *Humicola* were only related to “saprotroph”. Some non-dominant fungi including *Russula*, *Cortinarius*, *Lactarius*, *Tomentella*, *Laccaria*, *Cadophora*, and *Leptodontidium* were only related to “symbiotroph” (Fig. 6). It was also found that some unclassified fungi were matched into both “pathotroph” and “saprotroph” (Supplementary Table S3). More detailed information of trophic modes revealed that the highest abundance of subcategories was ‘plant pathogen’ (91.8%), followed by ‘undefined saprotroph’ (6.2%), ‘ectomycorrhizal’ (1.1%), ‘wood saprotroph’ (0.4%), ‘undefined root endophytes’ (0.3%), and ‘animal pathogen’ (0.26%) (Fig. 7 and Supplementary Table S4).

## Discussion

It has been demonstrated that macrofungal fruiting bodies could harbor a broad spectrum of microbes, especially the bacterial diversity and physiological role on host fungi were widely explored and reported in many researches [42, 43]. For instance, it was found that a variety of culturable bacteria were isolated from the fruiting bodies of *Suillus grevillei*, among which the isolates *Pseudomonas* sp. could remarkably promote the growth of host mycelia, whereas *Streptomyces* sp. possessed an inhibitory effect [44]. Xiang et al. (2017) revealed that two bacterial isolates, DJ35 and DY22 of *Pseudomonas* sp. from the *A. bisporus* fruiting body, promoted their host growth by the secretion of cellulase and indole-3-acetic acid [28]. Xu et al. (2021) reported that the metabolites of endophytic bacteria derived from mature fruiting bodies, especially strain Ld3 could ameliorate the quality of host *Lyophyllum decastes* by enhancing the non-volatile taste components including amino acid, protein, soluble sugar, etc. of host hyphae [45]. In our previous research, an associated bacterium named *P. fulva* SB1 from *Shiraia* fruiting body was found to provoke the yields of intracellular biosynthesis and extracellular excretion of medicinal perylenequinonoids of the host fungus [46]. In terms of endophytic fungi, the reports for them inhabiting in the soil or root systems of plant parasitized or infected by macrofungi were sufficient [47–49], but less information was regarding the fungal community associated with macrofungal fruiting bodies. In like wise, the community of associated fungi and their physiological roles in the fruiting bodies of *Sanghuangporus* were also still ignored.

To our best knowledge, this is the first report to present the fungal community and function in *Sanghuangporu* fruiting body by using a more advantageous culture-independent method of Illumina NovaSeq sequencing. The sequencing results furnished us with a more comprehensive data (865 OTUs) of the fungal diversity in *S. vaninii* fruiting body. As we expected, a large number of the ITS1 reads corresponded to the host fungal taxonomy (Fig. 4) [5], indicating an accurate sequencing analysis in this research. Most of the endophytic fungi belonged to the Ascomycota (14.8%) and Sordariomycetes (6.0%) (Fig. 4a, b), which was partly consistent with the results reported by Maurice et al. (2021) in forest fungal sporocarps [26]. Among the fungi, the order of Mortierellales was always related to long-lived fungal sporocarps, whereas Agaricales was related to short-lived ones [26, 50], indicating the detected endophytic fungi may be involved in the regulation of growth cycle of the host *S. vaninii*. Cladosporiales, Eurotiales, Helotiales or Hypocreales as the most abundant orders were either isolated or sequenced in several fungal sporocarps and xylarialean fruiting bodies [26, 51]. It was mentioned that the phylum of Sordariomycetes and the family of Chaetomiaceae were usually found in the compost of cultured mushroom [52], indicating some endophytic fungi may stem from the cultivation substrate of *S. vaninii*. The relative abundances of non-host ITS1 reads were dominated by five genera including *Mortierella*, *Cladosporium*, *Fusarium*, etc. (Fig. 4e), which were usually isolated and identified from the mushroom or macrofungal fruiting body but lack of reports as the members of fungal community [49, 53]. Hence, we hope that fungal community report of *Sanghuangporus* fruiting body can provide researchers a hint to pay attention to the endophytic fungi when cultivating a valuable macrofungi on a large scale.

Our research attempted to address the potential function of endophytic fungi in cultivated *Sanghuangporus* for the first time (Figs. 6 and 7 and Supplementary Table S2-S4). It was supposed that the most dominated *Phellinus* (corrected to *Sanghuangporus*) of the host fungi could make full use of the surrounding nutrition by decomposing the cultural substrate or attacking their tree hosts such as *Moru* and *Populus* [54, 55] for the massive growth of the fruiting body, because the genus of the host fungi was only annotated to the ‘plant pathotroph’ (Fig. 7 and Supplementary Table S4). Meanwhile, the non-host fungi were classified into the “pathotroph” type such as *Phlyctochytrium*, *Botryotinia*, *Aspergillus*, etc. (Fig. 6 and Supplementary Table S3), which could uptake nutrition by attacking host cells so they are expected to have adverse impacts on other members of the fungal community structure [56]. In addition, the “saprotroph” mode possessed the highest proportion (53.2%) of endophytic fungi, indicating *Rhizophlyctis*, *Trichocladium*, *Botryotrichum*, etc. (Fig. 6 and Supplementary Table S3) could obtain nutrition by degrading dead cells of the host fungi [57]. It was noteworthy that the second highest proportion (32.2%) of “symbiotroph” group of endophytic fungi including *Russula*, *Tomentella*, *Cadophora*, etc. were identified in fungal community structure (Fig. 6 and Supplementary Table S3), which may be involved in the growth, development, metabolism, and quality of hosts [58, 59] as well as bring a feasible idea for the increased production of *Sanghuangporus* via a coculture strategy of associated microbes with host fungus. As reported by Yurkov et al. (2012) [60], the Basidiomycetous yeast strains isolated from *Paxillus* and *Xercomus* fruiting bodies were able to secret some volatile compounds influencing the growth and development of mycoparasitic fungus. And our previous study demonstrated that the endophytic *P. fulva* could induce mass sporulation and photosensitizer production of host fruiting bodies of *Shiraia* [46].

## Conclusion

In summary, due to the microbes have important influences on the growth, development and active metabolite biosynthesis of hosts, we successfully analyzed and characterized the community composition and trophic mode diversity of fungi associated with the fruiting body of *S. vaninii* for the first time by high-throughput sequencing technique in this paper. Although the specific impacts and mechanisms of endophytic non-host fungi on the growth and metabolism of host-fungus *S. vaninii* need further investigation, the present work provided fundamental data for the active microbial excavation of associated sources. Since microbe’ co-culture inspired by the natural microbial community structure is becoming one of “One Strain, Many Compounds (OSMAC)” strategies to enhance chemodiversity [61, 62], we believe that the production and quality of macrofungus and its active metabolite in a large-scale culture of *Sanghuangporus* may be improved tremendously by its non-host endophytes in the future.

## Electronic supplementary material

Below is the link to the electronic supplementary material.

**Supplementary material 1: Table S1.** Taxonomic classification of OTUs detected from *Sanghuangporus vaninii* fruiting body with total ITS rDNA read numbers. **Table S2.** The fungal OTUs associated with *S. vaninii* fruiting body were annotated into trophic modes. **Table S3.** Trophic modes annotation of associated fungi at genus level. **Table S4.** Trophic modes and their guilds annotation of fungi associated with *S. vaninii* fruiting body.

## Data Availability

The datasets generated during the current study are available from the corresponding author on reasonable request and the raw sequences data have been submitted to the NCBI under accession No. PRJNA820174.

## Declarations

**Consent for publish** Not applicable.

**Author details**.

^1^ College of Life Sciences, Northwest Normal University, Lanzhou 730070, Gansu, China.

Received: 5 October 2022.
